# The Kneeling Isometric Plantar Flexor Test: Preliminary Reliability and Feasibility in Professional Youth Football

**DOI:** 10.3390/jfmk8040164

**Published:** 2023-11-30

**Authors:** John J. McMahon, Nicholas J. Ripley, Paul Comfort, Francisco Javier Robles-Palazón, Jack T. Fahey, Andrew J. Badby, Christopher Bramah

**Affiliations:** 1 Centre for Human Movement and Rehabilitation Research, University of Salford, Salford M6 6PU, UK; n.j.ripley@salford.ac.uk (N.J.R.); p.comfort@salford.ac.uk (P.C.); f.palazon@salford.ac.uk (F.J.R.-P.); j.t.fahey@salford.ac.uk (J.T.F.); a.j.badby@edu.salford.ac.uk (A.J.B.); c.a.bramah@salford.ac.uk (C.B.); 2 Centre for Exercise and Sport Science Research, Edith Cowan University, Joondalup, WA 6027, Australia; 3 Department of Physical Activity and Sport, Faculty of Sport Sciences, Campus of Excellence Mare Nostrum, University of Murcia, 30720 Murcia, Spain

**Keywords:** soleus, calf, maximal strength, force plate, soccer, injury

## Abstract

Calf injuries are common in professional football; thus, the establishment of reliable and time-efficient methods of measuring the peak force capabilities of the plantar flexors with equipment that is accessible to football practitioners is valuable. In this study, we determined the preliminary reliability and feasibility of a new test, termed the kneeling isometric plantar flexion test (KIPFT), for footballers. Twenty-one male youth footballers (age = 17.8 ± 1.1 years, height = 182 ± 5 cm, weight = 77.6 ± 5.9 kg) from English League One football clubs completed three trials of the KIPFT on a wireless force plate at the end (2022–2023) and start (2023–2024) of the season. The within-session reliability of the peak force (relative to body weight) was good–excellent for both limbs and both occasions. On average, performance of the KIPFT took just over 1 min per limb and ~2 min to set up. The peak force values were larger for the non-dominant limbs only at the start versus the end of the season, but there were no between-limb differences. From these results, it was determined that (1) the KIPFT is feasible, (2) a minimum of 32 footballers would be required to establish its between-session reliability with ≥80% statistical power and (3) large-cohort normative data for the KIPFT may be best collected at the start of the football season.

## 1. Introduction

Injuries to the calf complex, which comprises the soleus and gastrocnemius, represent a significant burden in sports, accounting for up to 13% of all football-related muscle injuries, with an injury incidence of 0.32 per 1000 h of football exposure among male footballers [1]. The soleus is the most frequently injured of all calf muscles [2], with footballers’ return to play time widely variable and injury recurrence reported to occur in more than 13% of cases [1,3]. The high injury and reinjury rates may in part be influenced by the high physical demands placed on the soleus during sport-specific tasks. During running, for example, the soleus is primarily responsible for the vertical support and acceleration of the center of mass, via plantar flexion, encountering external forces ranging from 1.5 times the body weight (xBW) to 2.4 xBW [4,5]. The contribution of the soleus, in conjunction with the gastrocnemius, to sport-specific tasks is also observed during sprint acceleration [6], deceleration [7] and change-in-direction performance [8], with its capacity to provide support and decrease strain on the anterior cruciate ligament [9]. Considering the high force demands placed on the calf musculature during sport-specific tasks, there is a need for feasible and reliable methods of assessing its force-generating qualities.

Half of surveyed strength and conditioning coaches working in football use force plates to regularly assess the force-generating capabilities of their athletes [10]. Force plates have only been used to assess isometric plantar flexion force in studies published since 2022 [11,12,13,14,15,16,17,18,19,20]. Only one study involved football players and included the seated isometric plantar flexor test (SIPFT) to determine its reliability of quantifying peak force [20]. The limitations of that study included that (1) the test was performed bilaterally, meaning that maximal force generation from either limb would likely not have been obtained [21]; (2) a soft foam pad was included between the anterior thighs and the metal bar to promote comfort, but this would have compressed during maximal force generation, thus reducing the peak force values measured and creating joint movement (no longer isometric); and (3) the statistical procedures were incorrect (i.e., the authors performed a Pearson correlation test, which is inappropriate for reliability determination [22], and calculated the minimal detectable change values incorrectly, leading to ~94% underestimation). Additionally, using a standard chair, bench or box during the SIPFT is unlikely to allow each athlete to obtain a horizontal thigh orientation (especially those who are shorter or taller than average) and may also lead to additional compression (and so joint movement) depending on its material properties (such as any cushioning on a gym bench or chair, for example).

Despite the abovementioned limitations of the SIPFT protocol utilized by Rhodes et al. [20], several other researchers have adopted a similar approach to assessing the isometric peak force capabilities of the plantar flexors [11,13,14,15,16,17,19]. An alternative approach to conducting the SIPFT, whereby a novel device specifically designed to assess the isometric force capabilities of the plantar flexors was validated [18] and used to assess a large cohort of rugby players, was recently published, thus providing normative reference values [12]. The strengths of the custom device and setup included the removal of a soft foam pad by using ratchet straps to fix the leg in position, thus removing the pad compression issue, and the test was conducted unilaterally, thus overcoming the bilateral strength deficit issue [21]. The limitations of these studies included the custom nature of the device, which would make replication of the test difficult for those who do not have access to it. Additionally, the authors placed the participants of these studies in full ankle dorsiflexion when they conducted the test to allow each participant to attain peak force generation. However, the results of a previous study have demonstrated that maximal plantar flexor torque occurs at almost full ankle dorsiflexion [23]. Additionally, while only a maximum angle of 20 deg. of dorsiflexion was tested, the difference in peak torque for 15 deg. or 20 deg. of dorsiflexion was negligible (170.2 ± 7.5 vs. 169.3 ± 7.1 Nm, respectively), suggesting that testing beyond this dorsiflexion angle would not lead to further increases in peak torque [23]. Furthermore, interindividual differences and intraindividual fluctuations in peak dorsiflexion over time may affect the compilation of normative data and the longitudinal monitoring of peak isometric plantar flexor force, respectively.

To overcome the abovementioned issues with the SIPFT, the authors of the present study created a potential solution in the form of the kneeling isometric plantar flexor test (KIPFT), conducted with commercially available force plates, given that they are regularly used by football practitioners [10]. Finding the preliminary reliability of any new test is one step in determining its feasibility and potential utility. If a test is not reliable within the same testing session, then it is unlikely to be reliable between testing sessions, which presents an issue when trying to monitor changes in the test scores. Additionally, to be able to estimate the required sample size for a full-scale reliability study, an estimate of the expected intraclass correlation coefficient (ICC) value is needed [24]. Another consideration is the feasibility (e.g., equipment and efficiency) of the KIPFT when conducting it with a full squad of footballers, as the time allocation for sports science and medicine staff to conduct testing is often limited. Even if the KIPFT is reliable, it is not useful for football researchers and practitioners if it cannot be easily implemented in practice. Lastly, quantifying the peak force values for the KIPFT during the in-season and pre-session periods will inform when it might be best to conduct a large-scale normative data study that can be used to inform practitioners who work in football of the typical KIPFT scores for healthy, injury-free players. Such normative data can then be used to set training benchmarks and guide players who sustain calf muscle injuries back to full-time training and competition.

The aims of this preliminary study were to (1) determine the within-session reliability of the KIPFT to inform the sample size required for a full-scale between-session reliability study involving professional footballers, (2) report the durations of key elements of the KIPFT to determine the feasibility of the test when conducted within authentic football environments and (3) compare the peak force values for the KIPFT during the in-season and pre-session periods to inform when might be the best period of the football season to conduct a normative data study.

## 2. Materials and Methods

### 2.1. Subjects

Twenty-one male youth football players from two different “category three” football academies (from League One football clubs in England) participated in this study (age = 17.8 ± 1.1 years, height = 182 ± 5 cm, weight = 77.6 ± 5.9 kg). They were classified as tier 3 (i.e., highly trained/national-level) participants [25]. The testing took place in May 2023 (end of season, PRE) and June 2023 (start of pre-season, PST). Testing was conducted at the same location (in a gym setting) and at approximately the same time of day (between 10:00 and 13:30) for each club and testing occasion, with any strenuous physical activity avoided in the previous 48 h. Informed consent was gained prior to testing, and this study was pre-approved by the authors’ institutional ethics committee (University of Salford: reference number 2090).

### 2.2. Tools

For the KIPFT, a single force plate (Hawkin Dynamics Inc., Portland, ME, USA) was placed on a portable isometric rig. The force plate hardware had been recently validated against industry gold-standard force plates during two high-force tasks [26]. The force plate was zeroed between each participant’s trials and sampled at 1000 Hz.

### 2.3. Procedure

Each participant knelt, with their non-tested leg placed posteriorly to the force plate and their knee on top of part of a hard-form surrounding that was the same height as the force plate. The non-tested thigh was vertical and in series with the hips, upright torso and head. The foot of the tested leg was placed on the force plate, with the femoral condyles positioned anteriorly and the malleoli positioned posteriorly to the uprights of the isometric rig, resulting in a diagonally orientated shin (with around 20 deg. of ankle dorsiflexion) and a horizontal thigh (i.e., 90 deg. of hip flexion). A previous study has shown that maximal plantar flexor torque occurs at almost full ankle dorsiflexion, with a maximum angle of 20 deg. of dorsiflexion tested [23]. Furthermore, the dorsiflexion range of motion is restricted when the knee is extended [23], and knee flexion also increases the contribution of the soleus to total plantar flexor force production [27], hence its inclusion.

**Figure 1:**
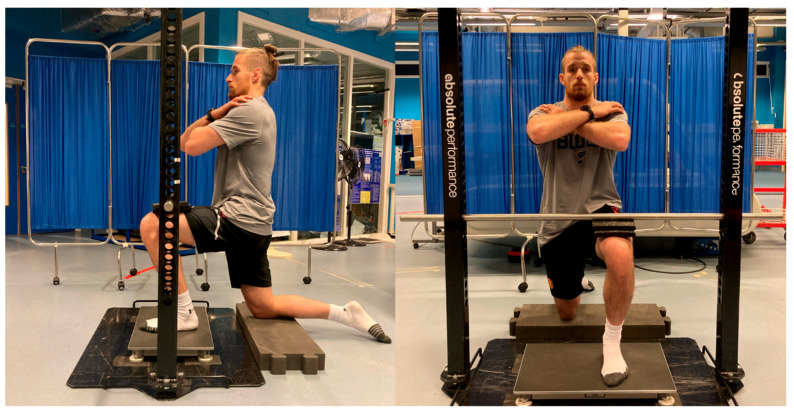
The sagittal (**left** image) and frontal (**right** image) view of the kneeling isometric plantar flexor test to illustrate the equipment and participant positioning.

Each participant performed the test while wearing only socks to account for the potential confounding influence of different footwear types on the resultant force data (e.g., compression of the shoe sole). Offcuts of hard rubber gym flooring were then placed between the anterior thigh of the tested leg, just proximal to the patella, and the bar of the isometric rig. The testers positioned the bar within the portable isometric rig as closely to the anterior thigh as possible and then used one or two offcuts of flooring to effectively “jam” the participant’s lower leg between the bar of the isometric rig and the force plate. The participant would then place their arms across their chest to limit extraneous movement. Two sub-maximal practice attempts were performed, and the participant was then instructed to push their knee up into the bar of the isometric rig as fast and hard as possible for 3–5 s for the maximal-effort trials. Three maximal-effort trials from each limb were recorded for each participant, with the limb order randomized between participants, interspersed with around 30–60 s of rest. A figure that illustrates the setup of the KIPFT is shown in Figure 1.

The force–time data were acquired via Hawkin Dynamics Inc. software (app version 8.6.1), which operated via an Android tablet that was connected to the force plate via Bluetooth. The force–time data were automatically low-pass filtered, with a 50 Hz cut-off frequency [28]. Peak force was also automatically analyzed by identifying the peak instantaneous vertical force applied during the 3–5 s push. A previous study reported that the peak force calculated with Hawkin Dynamics Inc. software (app version 8.6.1) for an isometric mid-thigh pull test was within 1 N (<0.1%) of that estimated with gold-standard data analysis procedures [29]. The data were exported from the Android tablet via Wi-Fi to the Hawkin Dynamics Inc. cloud server and later downloaded as a .csv file for management in Microsoft Excel. The peak force values were ratio-scaled to body weight (BW) in Microsoft Excel. The BW, which was recorded as the mean force during 1 s of standing completely upright and still on the force plates (in the same sessions as the KIPFT), was taken from the Hawkin Dynamics Inc. software (app version 8.6.1). To inform the feasibility of the KIPFT, the recorded duration between trials, the total test duration per limb, the setup duration and the duration of maximal-effort force production were identified from the data collection timestamps.

### 2.4. Statistical Analysis

A two-way mixed-effects model (absolute agreement, average measures) intraclass correlation coefficient (ICC), along with the upper and lower 95% confidence intervals (CI_95_), were used to determine the relative (i.e., rank-order) test–retest reliability, with <0.5, 0.5–<0.75, 0.75–0.90 and >0.90 (based on the upper CI_95_ of the CV% estimate) interpreted as poor, moderate, good and excellent relative reliability, respectively [30]. The within-subject coefficient of variation percentage (CV%) was calculated via the root mean square approach. Due to the relatively small sample size, the upper and lower CI_95_s of the CV% were calculated based on a T-distribution. Specifically, the standard error was first calculated by dividing the standard deviation of the CV% by the square root of the sample size. The lower and upper 95% CIs for the CV% were then calculated as the CV% means minus and plus a standard error of 2.064 [31], respectively, and expressed as percentages. CV%s of ≤10% and ≤5% have been used as indicators of acceptable reliability in previous, similar studies [32,33]. Due to a lack of consistency across those previous studies and to provide the qualitative scale that is the same as what was applied to the ICC outputs, the <5%, 5–10%, >10–15% and >15% thresholds (based on the upper CI_95_ of the CV% estimate) were considered to represent excellent, good, moderate and poor reliability, respectively. The data were normally distributed according to Shapiro–Wilk test results. Peak force values were compared within and between the limbs at each time point via paired *t*-tests. Each limb was classified as a dominant limb (DL) or non-dominant limb (NDL), with the former identified based on the participant’s preferred limb with which to kick a football [34]. Effect sizes were calculated using Hedges’ *g* method, providing a measure of the magnitude of the differences in each variable noted between time points, and were interpreted as trivial (≤0.19), small (0.20 to 0.49), moderate (0.50 to 0.79) or large (≥0.80) [35]. All data were analyzed with the Statistical Package for Social Sciences (SPSS version 26.0; SPSS Inc., Chicago, IL, USA), with statistical significance accepted at *p* ≤ 0.05.

## 3. Results

The relative reliability (i.e., ICC) of the peak force was excellent for both limbs and both time points (Table 1). The absolute reliability (i.e., CV%) of the peak force was excellent for both limbs and both time points, except for the DLs at the end of the season, for which the upper CI_95_ was classified as good. There were no significant or meaningful differences in peak between the NDL and DL peak force values at either time point (*p* = 0.306–0.808; *g* = 0.05–0.22). The NDL peak force did, however, significantly increase by a moderate amount between the end of the season and the start of pre-season testing (*p* = 0.007; *g* = 0.64 (95% CI range = 0.17–1.08)). The DLs showed a small but non-significant change from the end of the season and the start of pre-season testing (*p* = 0.106; *g* = 0.36 (95% CI range = 0.07–0.78)).

**Figure 2:**
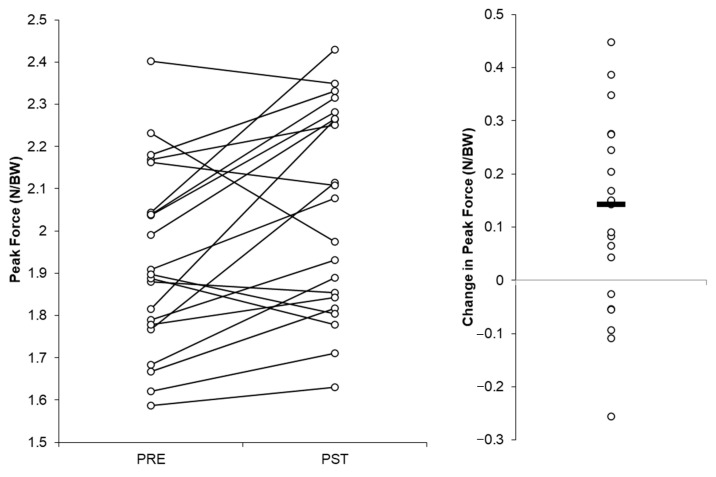
Peak force values and changes in peak force at/from the end of the 2022–2023 season (PRE) to the start of the 2023–2024 season (PST) for the non-dominant legs.

**Figure 3:**
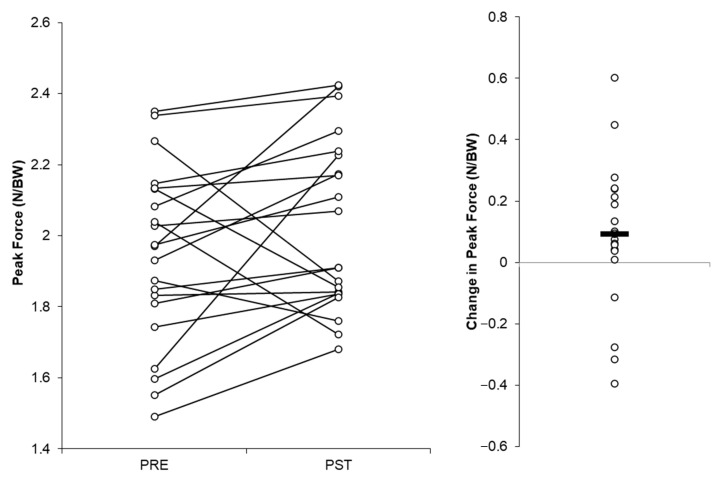
Peak force values and changes in peak force at/from the end of the 2022–2023 season (PRE) to the start of the 2023–2024 season (PST) for the dominant legs.

The individual players’ peak force values recorded at the end of the season and the start of pre-season testing and the mean changes between these occasions are visually presented in Figure 2 and Figure 3. Six and four of the 21 players produced larger peak force values at the start of the pre-season testing vs. the end-of-season testing with the NDLs and DLs, respectively (Figure 2 and Figure 3).

The recorded duration between trials, the total test duration per limb, the setup duration between limbs and the duration of maximal-effort force production (i.e., the pushing effort) during the KIPFT are reported in Table 2.

## 4. Discussion

One aim of this preliminary study was to determine the reliability of the KIPFT during the football in-season and pre-season phases. While the ICC and CV% values were marginally better at the start of the pre-season versus the end of the season, they were practically the same from an interpretation standpoint, suggesting that within-session peak force values during the KIPFT should be reliable at either phase of the season (Table 1). Football research is generally plagued by small sample sizes with insufficient statistical power [36]; moreover, most sport-science-based reliability studies are conducted without prior sample size estimation, which can lead to erroneous results [24]. Using the lowest ICC value from this study, of 0.902 (based on the lowest lower-bound CI_95_ in Table 1), and based on a hypothesis-testing approach with a minimum acceptable ICC of 0.75 [30], an alpha level of 0.05 and two testing occasions (i.e., test–retest design), 32 or 42 participants would be needed to achieve statistical power of 80% and 90%, respectively [24]. Thus, a future research priority is to determine the test–retest reliability of the peak force measurement during the KIPFT with a minimum of 32 footballers involved. This will allow for the identification of football-specific minimal changes in KIPFT peak force values that then can be applied to the longitudinal monitoring of KIPFT peak force by football researchers and practitioners. While seated and standing isometric plantar flexor tests have been shown to be reliable in assessing the force capabilities of ballet dancers and recreationally active participants [19], the one study to explore the test–retest reliability of the SIPFT with footballers used an incorrect statistical approach [20]; thus, the test–retest reliability of any isometric plantar flexor test conducted with professional football cohorts remains unknown.

Force plates are now being applied in clinical settings [37]. The bilateral version of the SIPFT, performed with a force plate, has been used in football as part of the return of a player to the sport following lateral ankle reconstruction [38]. As mentioned earlier, calf muscle injuries are also common in professional football [2], with widely variable return-to-sport times [1,3]. The mean KIPFT peak force range, from 1.93 to 2.05 xBW, is similar to the SIPFT normative values reported by Lee et al. [12], as attained by professional rugby union players. Knowing what the typical isometric peak force scores in these tests are for healthy, injury-free players is an important step in informing sports injury practitioners of the realistic benchmarks to which calf/ankle injured athletes can aspire. Interestingly, the mean KIPFT peak force values reported in this study are within the range of the external forces, of 1.5–2.4 xBW, encountered by the soleus during running [4,5]. This suggests that the external force demands placed on the soleus during running are close to and in many instances likely to exceed the maximal isometric force capabilities of the soleus. This highlights the importance of developing and accurately assessing peak force capabilities within rehabilitation and the return-to-sport process to ensure that physical qualities are suitably developed to cope with the biomechanical demands of sport-specific tasks.

Future work is needed to establish large, football-cohort-specific (e.g., age, level of play) normative data sets for the KIPFT to more accurately inform practitioners of expected peak force scores in injury-free players. Based on our findings in Figure 2 and Figure 3, it may be prudent to collect large-scale normative data during the early pre-season period, given that most players’ force values were larger at this timepoint, although this could represent a learning effect and is a limitation of this study. Additionally, exploring whether the KIPFT can provide any utility during the rehabilitation and subsequent return to sport of calf/ankle-injured players would be worthwhile. Moreover, although there were no significant or meaningful differences in peak force produced by the DLs or NDLs, whether this remains true for calf/ankle-injured athletes warrants exploration, in addition to whether rapid force production characteristics, such as the rate of force development, can be reliably measured during the KIPFT and differ between limbs.

Another aim of this preliminary study was to explore the feasibility (e.g., equipment and efficiency) of the KIPFT when conducting it with a full squad of footballers. As mentioned earlier, force plates are now regularly used by football practitioners [10], and even if practitioners do not have access to an isometric rig like the one shown in Figure 1, they could likely perform the KIPFT with a power rack and an overloaded barbell or Smith machine with a fixable bar. The average time to set the player and equipment in the correct position when switching between the first and second tested limb was just under 2 min (Table 2). Setting the bar of the isometric rig to the correct height for everyone’s first tested limb was the most time-consuming part of the testing, but once it was established, each individual remained in position for all three trials of their first tested limb, and the bar height did not need to be changed when their second limb was tested, so the setup for the second tested limb was much quicker. It is worth noting that the durations of the key events reported in Table 2 occurred naturally, meaning that the testers did not time these events while administering the KIPFT but rather judged them in the moment based solely on their experience of conducting this test as a part of pilot testing. If a player wanted longer time between trials, this was permitted; thus, the time taken between trials and to set up for the second tested limb could be reduced further if needed. Although precisely timing the key events of the KIPFT may have increased the peak force recordings and improved reliability, we wanted to authentically replicate the conditions within which football practitioners work (i.e., many players to test and minimal time available to test them), and the peak force reliability was still good–excellent with the approach we adopted. Based on the maximum duration of events reported in Table 2, it should take no more than 10 min per player to conduct the KIPFT.

Although this preliminary study shows that the KIPFT produced good–excellent reliability for peak force and can be conducted in professional football environments in a relatively time-efficient manner, it is worth remembering that we did not measure joint angles (knee and ankle) directly in this study. The authors of a recent study involving professional rugby union players being tested via the SIPFT (unilateral version) placed their participants in maximal dorsiflexion [12]. The peak dorsiflexion angle in professional youth soccer players was reported to be 36.1 ± 4.8 deg., but the values for the individual players ranged from 26.1 to 45.9 deg. [39]. Thus, the approximate 20 deg. of dorsiflexion attained by the players in this study is likely much below their maximal individual dorsiflexion angles; therefore, they may not have been in the most favorable position to produce maximal plantar flexor force, despite the findings of Sale et al. [23]. However, the limitation of placing each player in their peak dorsiflexion position is that if their ankle mobility changes over time, then so will the ankle angle at which they perform the KIPFT, which may make interpreting peak force changes more difficult, should the approach of Lee et al. [12] be taken. Thus, research into the influence of the ankle and knee angles on both the KIPFT and SIPFT (unilateral version) peak force values would be valuable, in addition to comparing the agreement between the peak force values obtained from these tests to determine whether can they be used interchangeably or compared within reasonable limits.

Lastly, during our pilot testing, we observed that the vertical shin and neutral ankle angle adopted during the bilateral version of the SIPFT [11,13,14,15,16,17,20] led to the ball of the foot being placed anterior to the contact point between the bar of the rig and the thighs. This is an issue when using single-axis-only force plates (i.e., those that record vertical forces only, which is typical of commercial-grade force plates), as the point of force application (i.e., the ball of the foot) is in front of the bar–thigh interface, which induces a large anterior–posterior force application that cannot be recorded with single-axis force plates. Due to placing the players in ankle dorsiflexion, the diagonal shin orientation adopted when performing the KIPFT, or, indeed, that would be expected if following the unilateral SIPFT protocol proposed by Lee et al. [12], overcame this issue by creating vertical alignment between the point of force application and the bar–thigh interface (Figure 1). For researchers and practitioners who have conducted or are currently conducting the SIPFT with a vertical shin orientation, quantifying the magnitude of the anterior–posterior force generation and thus the forces produced by the participant but omitted by single-axis force plates with a large cohort would be beneficial.

## 5. Conclusions

The within-session reliability of peak force (relative to body weight) during the KIPFT was good–excellent for both limbs and both test occasions, suggesting that the test would be reliable across three trials performed within the same session. From these preliminary results, it was determined that a minimum of 32 footballers would be required to establish the between-session reliability of the KIPFT peak force with ≥80% statistical power. On average, the KIPFT took just over 1 min per limb and ~2 min to set up, suggesting that it is an efficient test of plantar flexor maximal strength that can be conducted relatively quickly with entire squads of footballers, particularly if there is access to multiple force plate systems. Finally, the peak force values were larger for the non-dominant limbs at the start versus the end of the season, with no between-limb differences noted, suggesting that large-cohort normative data for the KIPFT may be best collected at the start of the football season.

## Figures and Tables

**Table 1:** Peak force (N/BW) and reliability statistics for each testing time point.

End of Season								
	Mean	SD	ICC	ICC_−95_	ICC_+95_	CV%	CV%_−95_	CV%_+95_
NDL	1.93	0.22	0.953	0.902	0.979	4.16	3.41	4.80
DL	1.94	0.25	0.965	0.928	0.985	4.20	2.92	5.18
**Start of Pre-Season**								
	Mean	SD	ICC	ICC_−95_	ICC_+95_	CV%	CV%_−95_	CV%_+95_
NDL	2.05 ^#^	0.24	0.979	0.957	0.991	3.02	2.28	3.62
DL	2.03	0.24	0.970	0.937	0.987	3.66	2.69	4.43

NDL = non-dominant leg, DL = dominant leg, ICC = interclass correlation coefficient, CV% = coefficient of variation percentage, −95 = lower 95% confidence interval, +95 = upper 95% confidence interval, SD = standard deviation. ^#^ = significantly different (*p* < 0.01) to the NDL value at end of season.

**Table 2:** Average and range of durations of key events during the KIPFT.

Variable	Mean	Min	Max
Intertrial rest duration (minutes:seconds)	00:36	00:16	01:09
Total within-limb test duration (minutes:seconds)	01:16	00:35	02:24
Total between-limb setup duration (minutes:seconds)	01:58	00:37	04:44
Maximal-effort push duration (seconds.milliseconds)	4.12	2.97	5.82

KIPFT = kneeling isometric plantar flexor test.

## Data Availability

The data presented in this study have been made openly available by the authors via FigShare at https://figshare.com/articles/dataset/Kneeling_Isometric_Plantar_Flexor_Test_Peak_Force_Data_xlsx/24460741 (uploaded on 30 October 2023).
